# Fabrication of ZnO/Red Phosphorus Heterostructure for Effective Photocatalytic H_2_ Evolution from Water Splitting

**DOI:** 10.3390/nano8100835

**Published:** 2018-10-15

**Authors:** Jiaqi Chen, Shaolong Huang, Yaojia Long, Jiahao Wu, Hui Li, Zhao Li, Yu-Jia Zeng, Shuangchen Ruan

**Affiliations:** Shenzhen Key Laboratory of Laser Engineering, College of Optoelectronic Engineering, Shenzhen University, Shenzhen 518060, China; 2160190414@email.szu.edu.cn (J.C.); nkhsl3313@163.com (S.H.); longyj1110@126.com (Y.L.); wujh83@mail2.sysu.edu.cn (J.W.); hui75401@gmail.com (H.L.); lizhao_520@163.com (Z.L.); scruan@szu.edu.cn (S.R.)

**Keywords:** photocatalysis, H_2_ evolution, red P, ZnO, heterostructure

## Abstract

Photocatalysis is a green technique that can convert solar energy to chemical energy, especially in H_2_ production from water splitting. In this study, ZnO and red phosphorus (ZnO/RP) heterostructures were fabricated through a facile calcination method for the first time, which showed the considerable photocatalytic activity of H_2_ evolution. The photocatalytic activities of heterostructures with different ratios of RP have been investigated in detail. Compared to bare ZnO, ZnO/RP heterostructures exhibit a 20.8-fold enhancement for H_2_ production and furthermore overcome the photocorrosion issue of ZnO. The improved photocatalytic activities highly depend on the synergistic effect of the high migration efficiency of photo-induced electron–hole pairs with the inhibited charge carrier recombination on the surface. The presented strategy can also be applied to other semiconductors for various optoelectronics applications.

## 1. Introduction

To solve the current energy crisis, H_2_ evolution from water splitting has been considered as one of the most promising methods for harvesting clean fuels. Since the pioneering work from Fujishima and Honda to induce the photo-assisted decomposition of water into H_2_ by using UV light in 1972, the photocatalytic properties of semiconductors have been studied in detail to directly convert solar energy into solar fuels [1,2,3]. Metal oxide semiconductors, such as TiO_2_ [4], WO_3_ [5], ZrO_2_ [6], SnO_2_ [7], CeO_2_ [8], ZnO [9], have been utilized as promising photo-catalyzers to generate H_2_ [10]. Among them, ZnO is nontoxic, low-cost and eco-friendly, and has been investigated for photoactivity [11,12,13]. However, the photocatalytic efficiency of ZnO is hindered by several shortages [14,15], including the high recombination rate of charge carriers, fatal photocorrosion [16], and the limited absorption of the solar spectra.

It is believed that heterojunction photocatalysts can promote carrier separation, which results in improved photocatalytic properties [17,18,19]. To enhance the photocatalytic performance of ZnO, constructing a heterojunction with other materials is a general strategy [20,21]. Recently, many metal-free elemental photocatalysts (Si, Se, P, S, B, Te) have received particular attention due to their good photoactivity [22]. Among them, red phosphorus (RP) is considered to be a promising candidate as it is a cost-effective and earth-abundant element [23,24]. Recently, black P/red P heterojunctions have been synthesized by an in-situ mechanical milling method, which shows enhanced photocatalytic activity for RhB dye degradation [25]. Xue et al. prepared a RP/C_3_N_4_ heterojunction for photocatalytic H_2_ production and CO_2_ conversion [26]. Moreover, RP and CdS have been constructed as heterostructure photocatalysts with enhanced photocatalytic H_2_ evolution activity [27]. However, heterostructure photocatalysts based on RP and ZnO have barely been studied.

In this study, ZnO/RP heterostructures have been prepared through a facile pressure-tight capsule calcination method, which shows enhanced photocatalytic H_2_ evolution performance and good photostability. By optimizing the composition and microstructure, the as-prepared nanoparticles exhibit excellent photocatalytic stability thanks to the coating of RP particles. The significantly enhanced H_2_ evolution rate is believed to result from the synergistic effect of the high migration efficiency of photo-induced electron–hole pairs with the inhibited charge carrier recombination in the interface.

## 2. Experiments

### 2.1. Synthesis of ZnO/RP Heterojunction Photocatalyst

Commercial ZnO (100 mg, Aladdin, Shanghai, China, AR 99.9%) was placed into SiO_2_ capsules and put into capsules with 1 mg, 5 mg, 10 mg, 15 mg of commercial RP (Aladdin, Shanghai, China), respectively. By adjusting the used amount of RP, the coverage density of the RP on the surface of the ZnO nanoparticle was controlled to obtain different ratios, which were marked as ZRP-X (X = weight percentage of added RP). Followed by vacuuming and sealing, the capsules were heated to 550 °C for 4 h at a heating rate of 5 °C/min, and then cooled down to room temperature naturally. For comparison, the pure ZnO power was also treated with the same procedure in the absence of red P.

### 2.2. Characterizations

X-ray diffraction (XRD) patterns were measured on instrument (D8Advance, Bruker, Karlsruhe, Germany) using Cu-K*α*-radiation. The acceleration voltage and the applied current were set as 40 kV and 40 mA, respectively. Scanning electron microscopy (SEM) was performed using a compact (SU70, Hitachi, Tokyo, Japan) with EDS mapping using Bruker XFlash 6I10 (XFlash 6I10, Bruker, Karlsruhe, Germany) at an accelerating voltage of 10 kV. Transmission electron microscopy (TEM) and high resolution TEM (HRTEM) analyses were measured on a JEM-2100&Aztec Energy TEM SP X-MaxN 80T (JEOL, Tokyo, Japan) using an accelerating voltage of 200 kV. The samples were prepared by applying a drop of ethanol suspension onto an amorphous carbon-coated copper grid and dried naturally. To figure out the surface chemical states, the X-ray photoelectron spectra (XPS) of the prepared photocatalysts were recorded on a Thermo Scientific ESCALAB 250Xi (ThermoFisher Scientific, Waltham, MA, USA). All spectra were calibrated to the binding energy of the adventitious C1s peak at 284.6 eV. UV-vis diffuse reflectance spectra (DRS) were recorded over the spectral range of 300–800 nm on a Perkin-Elmer Lambda 750 UV-vis spectrometer (Perkin Elmer, Waltham, MA, USA), using BaSO_4_ as a reflectance standard. Specific surface area (SBET) was determined with a surface area analyzer (Nova 2000e, Quantachrome, FL, USA) from the Brunauer-Emmett-Teller (BET) theory. Photoluminescence (PL) and Raman spectra were obtained on a HORIBA Jobin Yvon LabRAM HR (Horiba, Kyoto, Japan) at an excitation wavelength of 325 nm and 514 nm, respectively. The transient fluorescence decay spectra were measured by Edinburgh Instruments FLS920 fluorescence spectrophotometer (Edinburgh Instruments, Edinburgh, UK) using the 325 nm line of the Xe lamp as the excitation source.

### 2.3. Photocatalytic H_2_ Evolution Experiments

The photocatalytic H_2_ evolution experiments were performed using a reaction (CLE-SPH2N, Aulight, Beijing, China) cell connected to a closed gas circulation and evacuation system. In a typical procedure, 50 mg of the sample was dispersed in 100 mL of deionized water, with 10 vol% Triethanolamine (TEOA, Aladdin, Shanghai, China) as the hole’s sacrificial agent and 50 μL 3 wt% hydrochloroplatinic acid (ACS, 99.95%, Pt 37.5%, Alfa Aesar, Shanghai, China) as the co-catalyst. After being exposed to ultrasonic conditions for 5 min to get a homogeneous solution, the water splitting experiment was measured in a closed gas recirculation system equipped with a quartz reactor, connected to an evacuation pump and irradiated by a 300 W xenon lamp (CEL-HXF300, Aulight, Beijing, China) under one-sun light by using an AM 1.5 solar filter to obtain a measured intensity equivalent to standard AM 1.5 sunlight (100 mW/cm^2^). The light intensity was tested by an optical power meter (CEL-NP2000-10, Aulight, Beijing, China). The amount of H_2_ generated from the photocatalytic reaction was measured by a Techcomp GC 7920 gas chromatograph equipped with a TCD detector (Techcomp GC 7920, Shanghai, China) every 30 min. High-purity nitrogen gas was used as the carrier gas. In the long-time stability test, the reaction system was tested every 6 h of irradiation without adding extra TEOA or Pt.

## 3. Results and Discussion

**Figure 1:**
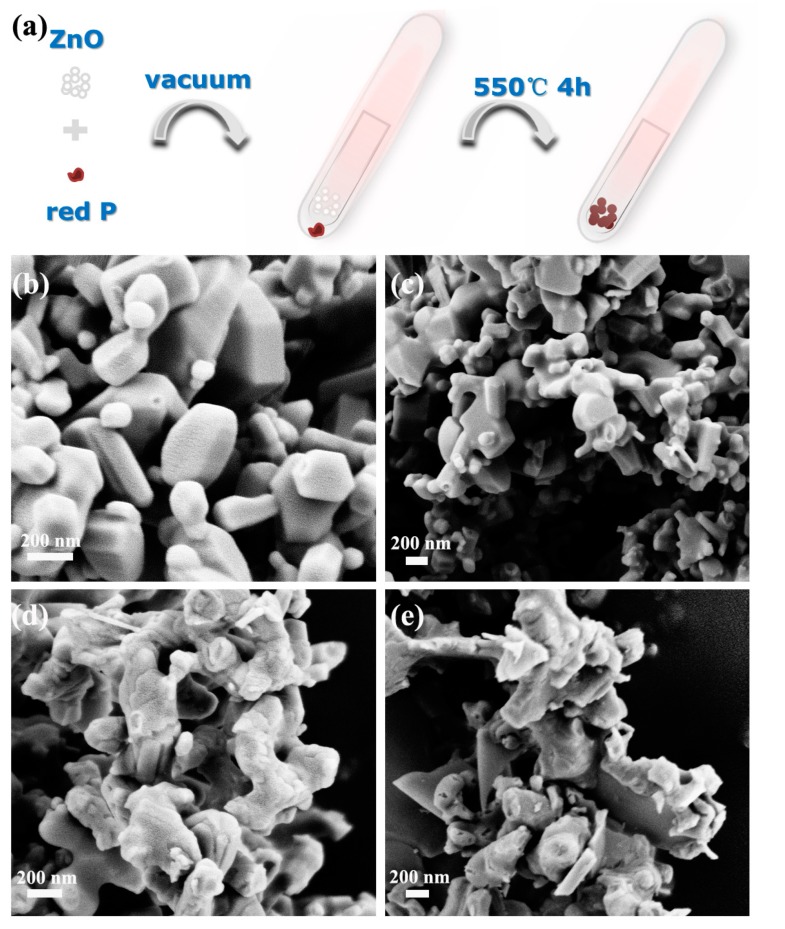
(**a**) Synthetic procedure for ZnO and red phosphorus (ZRP) heterostructure; (**b**–**e**) SEM images of ZnO-1, ZnO-5, ZnO-10 and ZnO-15.

**Figure 2:**
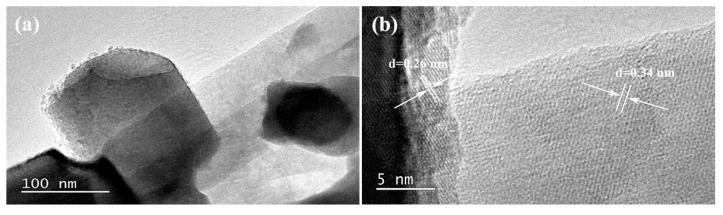
(**a**) TEM image of ZRP heterostructure; (**b**) HRTEM image of ZRP heterostructure.

**Figure 3:**
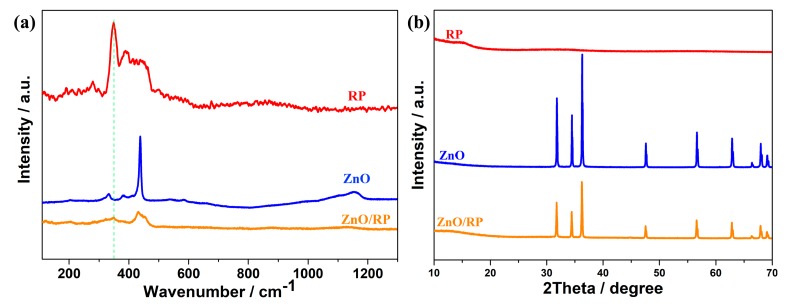
(**a**) Raman spectra of red P, ZnO and ZRP-5; (**b**) XRD patterns of the samples: ZnO, red P and ZRP-5.

As illustrated in Figure 1a, the heterostructure photocatalysts were prepared by calcining the mixture of RP and ZnO in vacuum. With the increase of the content of RP, the color of the heterojunctions becomes darker (Figure S1). The morphology and crystal phase of the as-synthesized samples were investigated with SEM, as shown in Figure 1b–e. It can be observed from Figure 1b,c that ZRP-1 and ZRP-5 exhibit a hexagonal structure of ZnO, which is consistent with the previous report [28]. On the other hand, the morphologies of ZRP-10 and ZRP-15 change as shown in Figure 1d,e, suggesting the increase of RP concentration in the ZnO/RP heterostructures. It is widely accepted that the activity of a photocatalyst is closely related to its morphology and crystallinity. Therefore, the heterostructures with different RP concentrations as well as different morphologies are expected to exhibit different performances of H_2_ evolution. The detailed structural information of ZnO/RP is shown in TEM images in Figure 2a. Figure 2b shows the HRTEM image of the heterostructure. The lattice spacing of 0.26 and 0.34 nm can be clearly observed, corresponding to the (101) plane of ZnO and (021) plane of RP, respectively [27]. These results confirm the formation of a heterostructure based on ZnO and RP. Furthermore, Figure 3a shows the Raman spectra of the ZnO, RP, and ZnO/RP heterostructures, where ZnO Raman modes are located at 350–550 cm^−1^ while the RP shows several well-defined modes in the 300–500 cm^−1^ region. For the ZnO/RP sample, a peak at 348 cm^−1^ related to RP is evident, indicateingthe existence of RP in the ZRP-5 heterostructure [29]. In Figure 3b, we show the XRD patterns of as-prepared samples. XRD patterns indicate that ZnO/RP heterostructures are well crystalline with a hexagonal structure, and the peaks centered at 31.7°, 34.5° and 47.5° can be indexed to the (100), (002) and (101) planes of hexagonal ZnO (PDF#80-0074). Due to the weak crystallization of RP, only one peak is found at 15° for RP corresponding to (102) planes (JCPDS card no. 44-0906) [27,29]. It can be considered that a proper amount of RP introduction does not change the phase structure of ZnO [30]. Compared to the pristine ZnO, no shift is detected in the ZnO/RP heterostructure, which confirms the formation of ZnO/RP heterojunction rather than RP doped ZnO.

**Figure 4:**
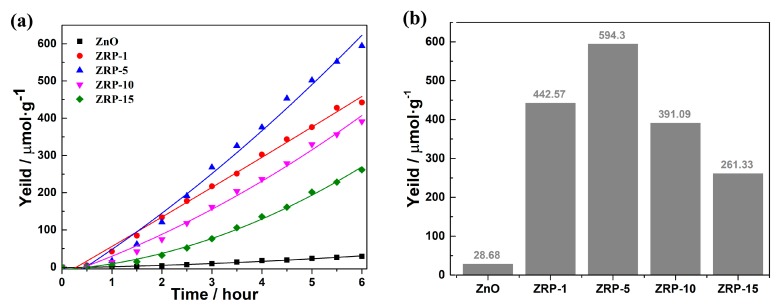
(**a**) Photocatalytic H_2_ evolution of the as-synthesized heterojunctions under AM 1.5 irradiation; (**b**) Photocatalytic H_2_ release yield in 6 h.

The photocatalytic H_2_ evolution rates of the samples (ZnO, ZRP-1, ZRP-5, ZRP-10 and ZRP-15) are measured under AM1.5 irradiation. As shown in Figure 4a, the H_2_ evolution amounts of ZnO, ZRP-1, ZRP-5, ZRP-10 and ZRP-15 are evaluated to be 28.68, 442.57, 594.30, 391.33, and 261.33 µmol g^−1^ in the first 6 h. The H_2_ evolution increases with the content of RP and reaches its optimal value when the proportion is 5 wt%. As shown in Figure 4b, ZRP-5 shows the highest photocatalytic activity in 6 h, which shows a 20.8-fold enhancement against the bare ZnO. Obviously, the amount of RP has a direct impact on the growth of the ZnO/RP [31]. Meanwhile, the low photoactivity of the pure ZnO can be attributed to its limited efficiency and the high recombination rate. The separation and transfer efficiency of the photo-generated charge carriers and recombination behavior of the photo-induced electron and hole are crucial to the photocatalytic H_2_ evolution.

**Figure 5:**
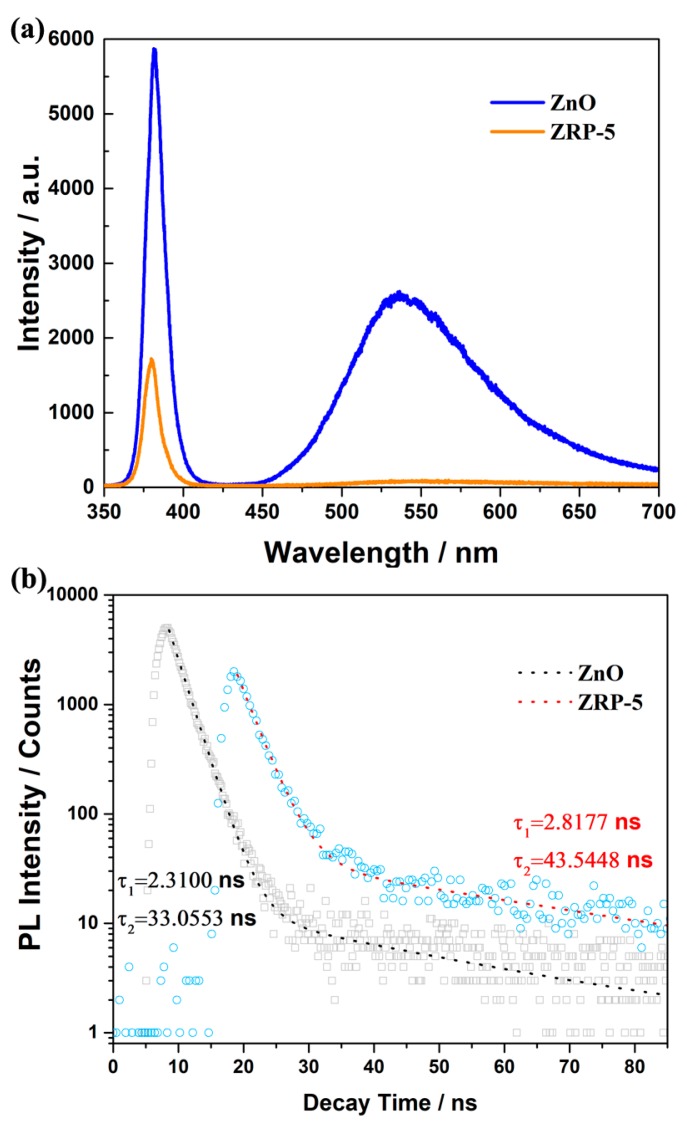
(**a**) Photoluminescence (PL) spectra of ZnO and ZRP-5; (**b**) Transient fluorescence decay spectra of ZnO and ZRP-5.

To investigate the separation and transfer efficiency of the photo-generated carriers over the ZnO/RP heterostructures, the PL spectra of the as-synthesized samples are shown in Figure 5a. It is known that the photo-induced electron in the conduction band will recombine with the hole at the valence band, leading to the emission of fluorescence [32]. Therefore, the quench of the PL spectra usually indicates the inhibition of the recombination for charge carriers, implying the separation of photo-induced electrons and holes. The pure ZnO shows two strong emission peaks: one is located at ~392 nm, corresponding to the near band gap excitonic emission, and the other is located at ~520 nm, attributed to the presence of singly ionized oxygen vacancies. In our ZnO/RP sample, both near band edge emission at 392 nm and defect-related emission at 520 nm decrease simultaneously. In addition, the photoactivity of the ZnO/RP sample increases as compared to the ZnO sample. Therefore, we believe that the decrease of PL is due to the formation of a heterostructure, which greatly increases the charge separation efficiency and reduces the recombination probability of photogenerated electron-holes [33,34]. Note that it has been reported that the defect-related emission at 475~625 nm disappears in the good-quality P-doped ZnO nanowires, which has been ascribed to the P doping effect instead of the formation of a heterojunction [35]. The results demonstrate an efficient separation of photo-excited electron–hole pairs between ZnO and RP, which is the main reason for enhancing the photocatalytic activity H_2_ evolution by using the ZnO/RP heterostructure.

Transient fluorescence decay spectra have also been used to illustrate the recombination efficiency of photo-induced carriers. The lifetime and the percentage of the charge carrier are summarized in Figure 5b. The decay time with τ_1_ (2.31 ns, 96%) and τ_2_ (33.05 ns, 4%) of the transient fluorescence are detected in the pure ZnO with 325 nm excitation. On the other hand, in the ZnO/RP heterostructure, both τ_1_ (2.81 ns, 78%) and τ_2_ (43.54 ns 22%) increase as compared with those for pure ZnO. This is because a portion of electrons in the conduction band (CB) of ZnO recombine with the holes in the valence band (VB) of RP, resulting in a decreased recombination of photogenerated electron–hole pairs in ZnO and extending the lifetime of holes in the VB of ZnO [36,37]. As confirmed, the fast decay component τ_1_ 2.31 ns of ZnO/RP is mainly assigned to the decreased lifetime of the charge recombination in the heterojunction due to the improved charge redistribution on the heterointerface [38]. Moreover, the lifetime of τ_2_ 43.54 ns, which is due to the prolonged charge recombination process in the ZnO/RP can maintain the photocatalytic activity and reduce the recombination of electron–hole pairs. As a result, the formation of new interaction generates more effective charge separation and improves the photocatalytic activity of H_2_ production.

**Figure 6:**
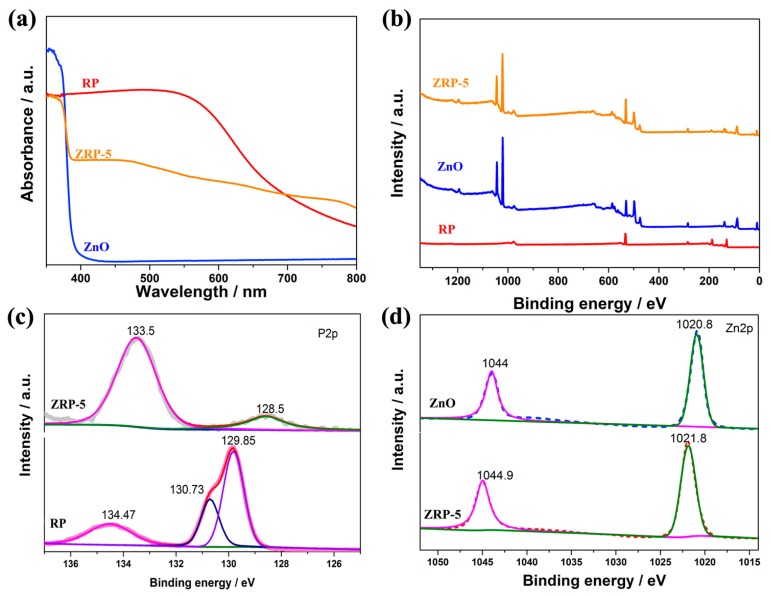
(**a**) UV-vis diffuse reflectance spectra of ZnO, red P and ZRP-5; (**b**) The XPS survey spectra of ZnO, red P and ZRP-5; (**c**) The XPS spectra P *2p* of ZRP-5 and red P; (**d**) The XPS Zn *2p* spectra of ZnO and ZRP-5.

The photocatalytic activities of semiconductors are closely related to their structure and the inner electron behavior; thus, the UV−vis absorption spectra were measured. Figure 6a exhibits UV-vis absorption features of ZnO, RP and ZRP-5. Accordingly, RP and ZnO show a fundamental absorption edge at 719 nm and 373 nm, respectively. The high absorption efficiency of these heterojunction photocatalysts will harvest more photons into the photocatalytic reaction process to enhance the photocatalytic activity [39]. As expected, the absorption edge of the heterojunction ZRP-5 exhibits a subtle red shift to the visible light region. According to the Kubelka–Munk rule, the bandgap of RP and ZnO is calculated to be 1.54 and 3.27 eV [25,40], respectively. The presence of RP enhances the light absorption for the ZnO/RP sample significantly in the region of λ > 400 nm, which is due to the formation of ZnO/RP heterojunction and the interfacial interaction [27].

XPS analysis was performed to investigate the chemical states of pure ZnO, ZRP-5 and RP, as shown in Figure 6b. The P 2*p* spectra of ZRP-5 and RP are shown in Figure 6c. For the RP, the spectrum shows that its surface is mainly composed of P^0^ atoms (129.8 eV) and a certain amount of P^5+^ atoms (134.47 eV) [32,41]. For ZRP-5, there are two peaks of P 2*p* located at 133.5 and 128.5 eV, which is consistent with P^5+^ and P^0^, respectively. Remarkably, the binding energy of P 2*p* shifts toward lower energies compared with that of the pristine RP, revealing that the charge transfer in these heterojunction photocatalysts. In Figure 6d, Zn 2*p* spectrum shows two strong peaks located at 1020.8 and 1044 eV for the pure ZnO. ZRP-5 it presents double peaks at 1021.8 and 1044.9 eV. These two peaks shift to the higher binding energies, indicating the strong interaction between ZnO and RP and the possible transfer of photogenerated charge carriers [32]. The XPS results reveal the strong interaction and chemical bonding between ZnO and RP, which are believed to result in the fast immigration of charge carriers and the separation of photo-excited electron–hole pairs.

To investigate the effect on the surface area and pore structure of ZnO/RP, the N_2_ adsorption–desorption isotherms were measured, and the textural parameters derived from the isotherms data are summarized in Table S1. The BET specific surface area of pure ZnO and ZRP-5 are 11.85 m^2^/g and 16.24 m^2^/g, respectively. However, we cannot find a direct correlation between photocatalytic H_2_ evolution activity between the BET surface area and pore volume, which excludes the possibility that the BET surface area and pore volume are the crucial factors for the improved photocatalytic activity [32].

For comparison, the photoactivity of the mechanical mixture of ZnO and RP was also investigated. Figure S2 displays the photocatalytic H_2_ production of the mechanical mixture sample, which shows negligible H_2_ evolution, even smaller that of pure ZnO, probably due to the shield effect [41]. The results highlight the important role the interface of the ZnO/RP heterostructure, which is crucial to the formation of electronic interaction and electron transfer. Note that no photocatalytic activity has been observed for the pure commercial red phosphorus used in this study. ZRP-X heterostructures show obviously increased H_2_ evolution rates due to the improved light absorption and the low recombination of photogenerated carriers resulting from the heterojunction structure [13]. However, with the increasing concentration of RP (>5 wt %) in the heterostructure, the photocatalytic reactivity appears to decrease. This might be attributed to the serious agglomeration of RP particles, leading to the blocking of the light absorption [27].

As a typical photocatalyst, the instability has been a critical issue for ZnO because of the photocorrosion [23]. Figure S3a shows the evolution curves of H_2_ in a cycling photocatalytic three times to test the stability of the heterojunction. The ZnO/RP heterostructure tends to be stable, and no quick decrease is observed after the test, showing the good photostability of the heterojunction due to the introduction of RP. In addition, the XPS patterns Figure S3b of ZRP-5 before and after 18 h irradiation are determined to investigate the structure stability, and no distinguishable change can be observed, providing evidence for the improved stability of heterojunctions.

In order to determine the photocatalytic mechanism of the heterostructure, the ZRP-5 sample is measured by valence band XPS, and the valance band minimums (VBMs) of the samples are determined in Figure S4. Both ZnO and ZRP-5 samples display a typical VBM at about 2.27 eV. However, compared with the pure ZnO, a small peak within the range of 0.5–2.0 eV is located at 1.2 eV for ZRP-5, which indicates that the VBM of RP is approximately located at 1.07 eV. Based on their bandgap values, the band alignment of the heterojunction belongs to type-I.

**Figure 7:**
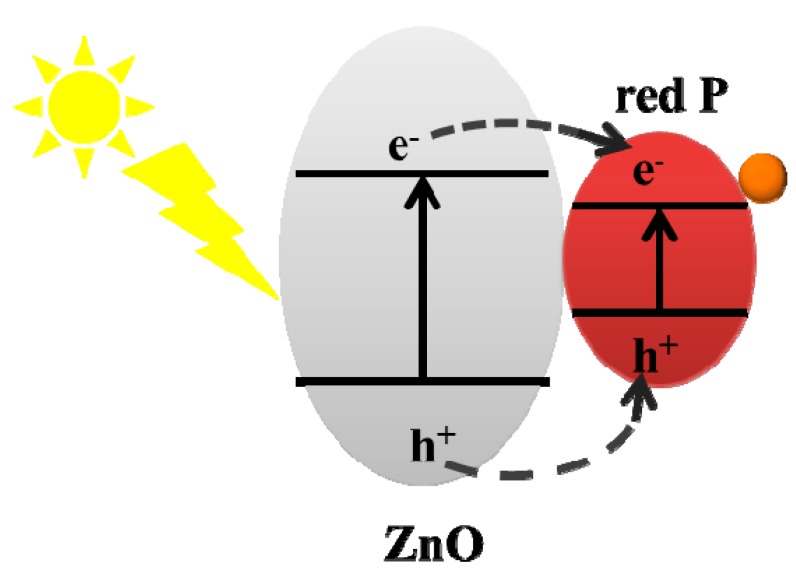
Type—band alignment of ZnO and RP.

The type-I heterojunction in Figure 7 is considered to be the possible mechanism to explain the improved photocatalytic activity [20]. For the type-I configuration in our work, under the light irradiation, electrons and holes will accumulate in the conduction band minimum (CBM) and VBM of RP, respectively. Therefore, efficient band alignment can be used to separate the charge carriers and reduce the carrier recombination, which results in significant enhancement of photocatalytic activity. Besides this, chloroplatinic acid was added before the photocatalytic reaction. Under AM 1.5 radiation, the Pt^+^ ions are reduced to Pt nanoparticles at the reduction sites. Therefore, the reaction site can be determined by the Pt distribution on the samples after the reaction. After the photocatalyst reaction, Pt is loaded on the RP as confirmed by the EDS mapping (Figure S5): this result indicates that the reduction reaction occurs on the RP site, which is consistent with the mechanism discussed above.

## 4. Conclusions

We have developed a novel kind of type-I ZnO/RP heterostructure photocatalyst through a simple calcination method under vacuum. Compared to the pure ZnO and red P, ZnO/RP heterostructures exhibit enhanced photocatalytic activity for H_2_ evolution and excellent photostability under AM1.5 light irradiation. As confirmed by PL and transient fluorescence spectra, the enhancement of water splitting to H_2_ evolution is believed to result from the rapid transfer and effective separation of photogenerated electrons and holes between the heterointerface of ZnO and RP. Thus, the increased charge carrier lifetime and the decreased recombination rate of the photogenerated electron–hole pairs both contribute to the enhancement of photocatalytic activity. This work not only demonstrates a photocatalyst based on ZnO/RP heterostructure, but also provides a simple strategy to construct heterojunctions that can also be applied to other semiconductors for optoelectronics applications.

## Supplementary Materials

The following are available online at http://www.mdpi.com/2079-4991/8/10/835/s1, Figure S1: Digital photograph of the heterostructure (A:ZRP-1, B:ZRP-5, C:ZRP-10, D:ZRP-15), Figure S2: The photocatalytic H2 production of red P, ZnO, and mechanical mixture (the content of red P is 5%), Figure S3: (a) The recycling H2 evolution reaction of ZRP-5 heterojunction. (b)The compared XPS pattern of ZRP-5 before and after irradiation, Figure S4: The valence band XPS of ZnO and ZRP-5, Figure S5: (a) SEM image of recycled ZRP-5 sample after water splitting photocatalytic reaction. (b–d) EDS mapping of O, Zn, P and Pt. (f) EDS spectra; Table S1: Specific surface area and pore volume of the samples.

## References

[1] Yu C., Yu J.C., Zhou W., Yang K. (2010). WO_3_ Coupled P-TiO_2_ photocatalysts with mesoporous structure. Catal. Lett..

[2] Ran J., Zhang J., Yu J., Jaroniec M., Qiao S.Z. (2014). Earth-abundant cocatalysts for semiconductor-based photocatalytic water splitting. Chem. Soc. Rev..

[3] Fujishima A., Honda K. (1972). Electrochemical photolysis of water at a semiconductor electrode. Nature.

[4] Yang Y., Liu G., Irvine J.T., Cheng H.M. (2016). Enhanced photocatalytic H_2_ production in Core-Shell engineered rutile TiO_2_. Adv. Mater..

[5] Jungwon K., Chulwee L., Wonyong C. (2010). Platinized WO_3_ as an environmental photocatalyst that generates OH radicals under visible light. Environ. Sci. Technol..

[6] Kambur A., Pozan G.S., Boz I. (2012). Preparation, characterization and photocatalytic activity of TiO_2_–ZrO_2_ binary oxide nanoparticles. Appl. Catal. B.

[7] Dodd A., McKinley A., Saunders M., Tsuzuki T. (2006). Mechanochemical synthesis of nanocrystalline SnO_2_–ZnO photocatalysts. Nanotechnology.

[8] Zhang N., Fu X., Xu Y.-J. (2011). A facile and green approach to synthesize Pt@CeO_2_ nanocomposite with tunable core-shell and yolk-shell structure and its application as a visible light photocatalyst. J. Mater. Chem..

[9] Li Y., Wang L., Liang J., Gao F., Yin K., Dai P. (2017). Hierarchical heterostructure of ZnO@TiO_2_ hollow spheres for highly efficient photocatalytic hydrogen evolution. Nanoscale Res. Lett..

[10] Khan M.M., Adil S.F., Al-Mayouf A. (2015). Metal oxides as photocatalysts. J. Saudi Chem. Soc..

[11] Lee K.M., Lai C.W., Ngai K.S., Juan J.C. (2016). Recent developments of zinc oxide based photocatalyst in water treatment technology: A review. Water Res..

[12] Zeng Y.J., Pereira L.M., Menghini M., Temst K., Vantomme A., Locquet J.P., Van Haesendonck C. (2012). Tuning quantum corrections and magnetoresistance in ZnO nanowires by ion implantation. Nano Lett..

[13] Zeng Y.J., Ye Z.Z., Lu Y.F., Lu J.G., Sun L., Xu W.Z., Zhu L.P., Zhao B.H., Che Y. (2007). ZnMgO quantum dots grown by low-pressure metal organic chemical vapor deposition. Appl. Phys. Lett..

[14] Ma D., Shi J.W., Zou Y., Fan Z., Ji X., Niu C. (2017). Highly efficient photocatalyst based on a CdS Quantum Dots/ZnO nanosheets 0D/2D heterojunction for hydrogen evolution from water splitting. ACS. Appl. Mater. Inter..

[15] Wang X., Liu G., Chen Z.G., Li F., Wang L., Lu G.Q., Cheng H.M. (2009). Enhanced photocatalytic hydrogen evolution by prolonging the lifetime of carriers in ZnO/CdS heterostructures. Chem. Commun..

[16] Zhang L., Cheng H., Zong R., Zhu Y. (2009). Photocorrosion Suppression of ZnO Nanoparticles via Hybridization with Graphite-like Carbon and Enhanced Photocatalytic Activity. J. Phys. Chem. C.

[17] Wang H., Zhang L., Chen Z., Hu J., Li S., Wang Z., Liu J., Wang X. (2014). Semiconductor heterojunction photocatalysts: Design, construction, and photocatalytic performances. Chem. Soc. Rev..

[18] Fujishima A., Zhang X., Tryk D. (2007). Heterogeneous photocatalysis: From water photolysis to applications in environmental clean up. Int. J. Hydrogen Energy.

[19] Low J., Cao S., Yu J., Wageh S. (2014). Two-dimensional layered composite photocatalysts. Chem. Commun..

[20] Low J., Yu J., Jaroniec M., Wageh S., Al-Ghamdi A.A. (2017). Heterojunction Photocatalysts. Adv. Mater..

[21] Wang Y., Wang Q., Zhan X., Wang F., Safdar M., He J. (2013). Visible light driven type II heterostructures and their enhanced photocatalysis properties: A review. Nanoscale.

[22] Hu Z., Shen Z., Yu J.C. (2017). Phosphorus containing materials for photocatalytic hydrogen evolution. Green Chem..

[23] Hu Z., Yuan L., Liu Z., Shen Z., Yu J.C. (2016). An elemental phosphorus photocatalyst with a record high hydrogen evolution efficiency. Angew. Chem. Int. Ed..

[24] Wang F., Ng W.K.H., Yu J.C., Zhu H., Li C., Zhang L., Liu Z., Li Q. (2012). Red phosphorus: An elemental photocatalyst for hydrogen formation from water. Appl. Catal. B.

[25] Shen Z., Sun S., Wang W., Liu J., Liu Z., Yu J.C. (2015). A black–red phosphorus heterostructure for efficient visible-light-driven photocatalysis. J. Mater. Chem. A.

[26] Yuan Y.-P., Cao S.-W., Liao Y.-S., Yin L.-S., Xue C. (2013). Red phosphor/g-C_3_N_4_ heterojunction with enhanced photocatalytic activities for solar fuels production. Appl. Catal. B.

[27] Shi Z., Dong X., Dang H. (2016). Facile fabrication of novel red phosphorus-CdS composite photocatalysts for H_2_ evolution under visible light irradiation. Int. J. Hydrogen Energy.

[28] Qi K., Yang J., Fu J., Wang G., Zhu L., Liu G., Zheng W. (2013). Morphology-controllable ZnO rings: Ionic liquid-assisted hydrothermal synthesis, growth mechanism and photoluminescence properties. CrystEngComm.

[29] Chang W.C., Tseng K.W., Tuan H.Y. (2017). Solution Synthesis of Iodine-Doped Red Phosphorus Nanoparticles for Lithium-Ion Battery Anodes. Nano Lett..

[30] D’Amato C.A., Giovannetti R., Zannotti M., Rommozzi E., Minicucci M., Gunnella R., Di Cicco A. (2018). Band gap implications on nano-TiO_2_ surface modification with ascorbic acid for visible light-active polypropylene coated photocatalyst. Nanomaterials.

[31] Gong C., Du J., Li X., Yu Z., Ma J., Qi W., Zhang K., Yang J., Luo M., Peng H. (2018). One-step acidic hydrothermal preparation of dendritic rutile TiO_2_ nanorods for Photocatalytic Performance. Nanomaterials.

[32] Ma D., Shi J.-W., Zou Y., Fan Z., Ji X., Niu C., Wang L. (2017). Rational design of CdS@ZnO core-shell structure via atomic layer deposition for drastically enhanced photocatalytic H_2_ evolution with excellent photostability. Nano Energy.

[33] Yu Z.B., Xie Y.P., Liu G., Lu G.Q., Ma X.L., Cheng H.-M. (2013). Self-assembled CdS/Au/ZnO heterostructure induced by surface polar charges for efficient photocatalytic hydrogen evolution. J. Mater. Chem. A.

[34] Moon S.C., Mametsuka H., Tabata S., Suzuki E. (2000). Photocatalytic production of hydrogen from water using TiO_2_ and B/TiO_2_. Catal. Today.

[35] Gao J., Zhao Q., Sun Y., Li G., Zhang J., Yu D. (2011). A Novel Way for Synthesizing Phosphorus-Doped Zno Nanowires. Nanoscale Res. Lett..

[36] Liu C., Zhang Y., Dong F., Reshak A.H., Ye L., Pinna N., Zeng C., Zhang T., Huang H. (2017). Chlorine intercalation in graphitic carbon nitride for efficient photocatalysis. Appl. Catal. B.

[37] Xu T., Zhang L., Cheng H., Zhu Y. (2011). Significantly enhanced photocatalytic performance of ZnO via graphene hybridization and the mechanism study. Appl. Catal. B.

[38] Yang C., Qin J., Xue Z., Ma M., Zhang X., Liu R. (2017). Rational design of carbon-doped TiO_2_ modified g-C_3_N_4_ via in-situ heat treatment for drastically improved photocatalytic hydrogen with excellent photostability. Nano Energy.

[39] Zhang Z., Huang J., Fang Y., Zhang M., Liu K., Dong B. (2017). A nonmetal plasmonic Z-Scheme photocatalyst with UV-to NIR-Driven photocatalytic protons reduction. Adv. Mater..

[40] Gao J., Zhang X., Sun Y., Zhao Q., Yu D. (2010). Compensation mechanism in N-doped ZnO nanowires. Nanotechnology.

[41] Jing L., Zhu R., Phillips D.L., Yu J.C. (2017). Effective prevention of charge trapping in graphitic carbon nitride with nanosized red phosphorus modification for superior photo(electro)catalysis. Adv. Funct. Mater..

