# Supplementary material for: Fabrication of ZnO/Red Phosphorus Heterostructure for Effective Photocatalytic H2 Evolution from Water Splitting
Source: Nanomaterials (Basel). 2018 Oct 15;8(10):835. doi: 10.3390/nano8100835 (PMC6215150; doi:10.3390/nano8100835)
Supplement: Supplementary file 1 [file nanomaterials-08-00835-s001.pdf]

Supporting Information

Fabrication of ZnO/Red Phosphorus Heterostructure for Effective Photocatalytic H_2 Evolution from Water Splitting

Jiaqi Chen, Shaolong Huang, Yaojia Long, Jiahao Wu, Hui Li, Zhao Li, Yu-Jia Zeng * and Shuangchen Ruan

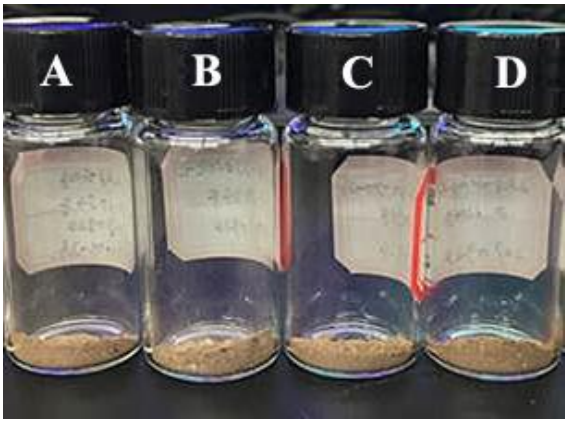

Figure S1. Digital photograph of the heterostructure (A:ZRP-1, B:ZRP-5, C:ZRP-10, D:ZRP-15).

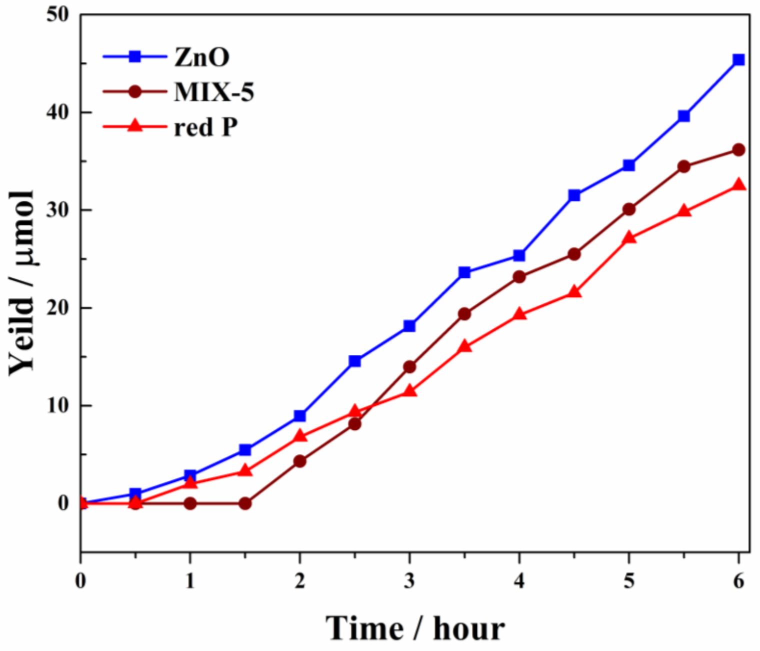

Figure S2. The photocatalytic H_2 production of red P, ZnO, and mechanical mixture (the content of red P is 5%).

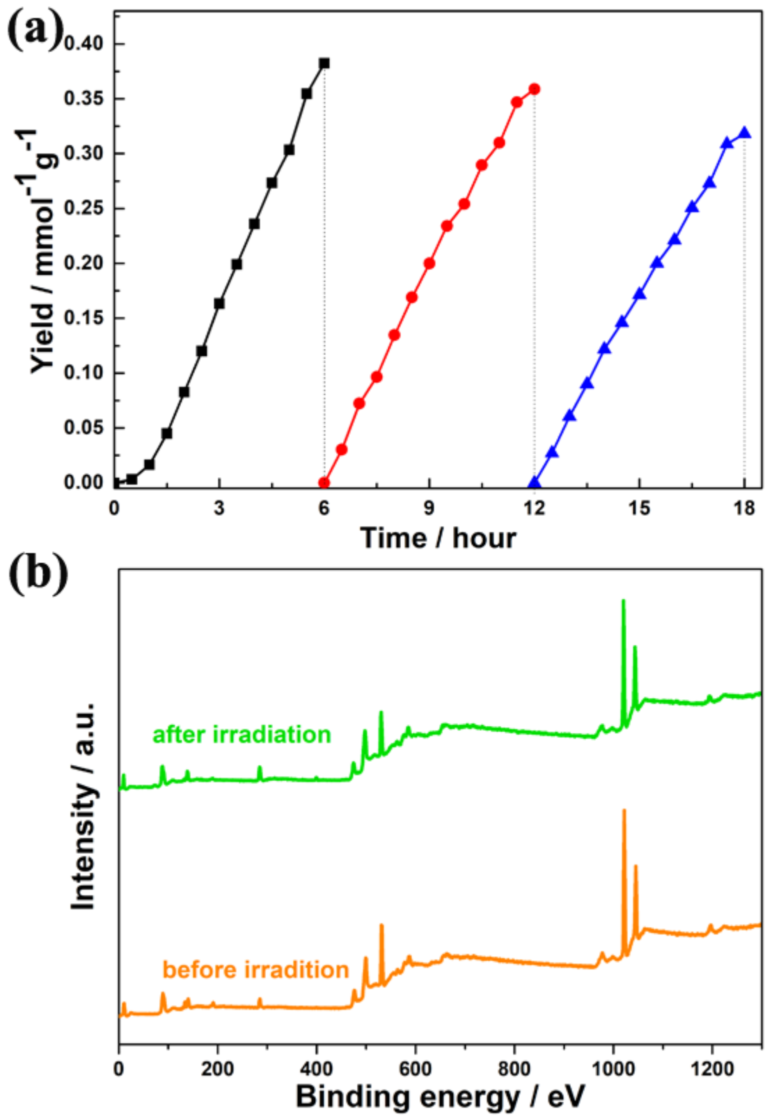

Figure S3. (**a**) The recycling H_2 evolution reaction of ZRP-5 heterojunction. (**b**) The compared XPS pattern of ZRP-5 before and after irradiation.

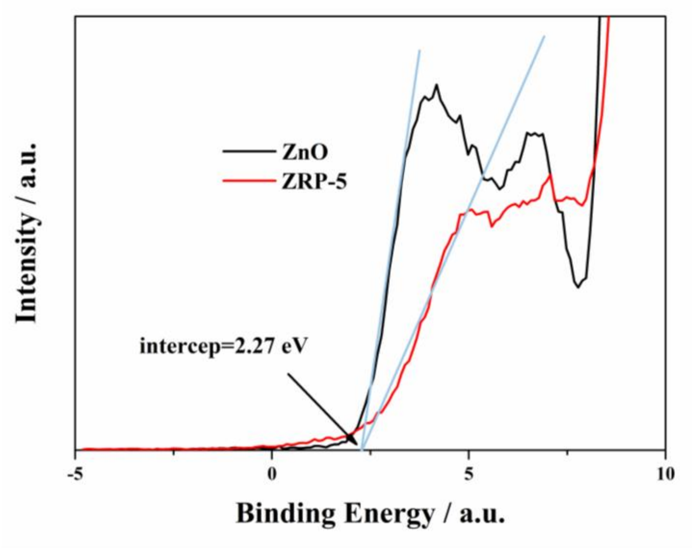

Figure S4. The valence band XPS of ZnO and ZRP-5.

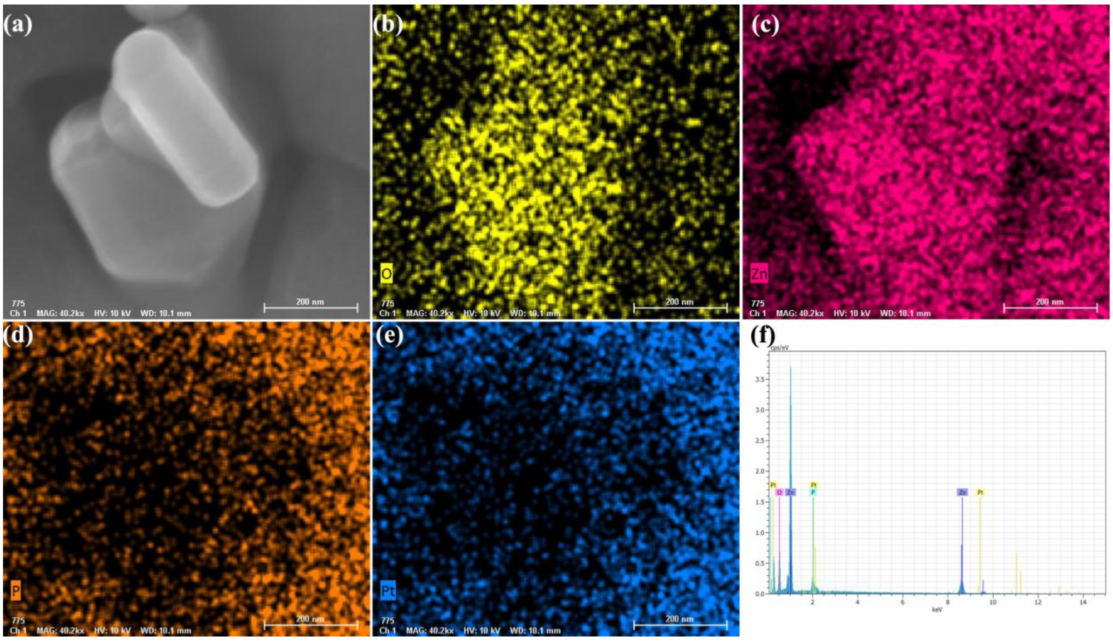

Figure S5. (**a**) SEM image of recycled ZRP-5 sample after water splitting photocatalytic reaction. (**b**–**d**) EDS mapping of O, Zn, P and Pt. (f) EDS spectra.

Table S1. Specific surface area and pore volume of the samples.

Samples	Pore volume/cm³/g	S_{BET}/m²/g
ZnO	0.0117 cm^3/g	11.8545 m^2/g
ZRP-5	0.0156 cm^3/g	16.2399 m^2/g
